# Co-occurrence of unhealthy lifestyle behaviours in middle-aged adults: findings from the Swedish CArdioPulmonary bioImage Study (SCAPIS)

**DOI:** 10.1038/s41598-024-71092-0

**Published:** 2024-10-01

**Authors:** Leonie Klompstra, Marie Löf, Cecilia Björkelund, Mai-Lis Hellenius, Lena V. Kallings, Marju Orho-Melander, Patrik Wennberg, Preben Bendtsen, Marcus Bendtsen

**Affiliations:** 1 Department of Health, Medicine and Caring Sciences, Linköping University, Linköping, Sweden; 2 Department of Biosciences and Nutrition, Karolinska Institutet, Stockholm, Sweden; 3 Primary Health Care/Department of Public Health and Community Medicine, Institute of Medicine, Sahlgrenska Academy, University of Gothenburg, Gothenburg, Sweden; 4 Research, Education, Development and Innovation, Primary Health Care, Region Västra Götaland, Sweden; 5 Department of Medicine, Karolinska Institutet, Stockholm, Sweden; 6 Department of Physical Activity and Health, The Swedish School of Sport and Health Sciences, Stockholm, Sweden; 7 Family Medicine and Preventive Medicine, Department of Public Health and Caring Sciences, Uppsala University, Uppsala, Sweden; 8 Department of Clinical Sciences, Lund University, Malmö, Sweden; 9 Department of Public Health and Clinical Medicine, Family Medicine, Umeå University, Umeå, Sweden; 10 Department of Medical Specialist in Motala, and Department of Health, Medicine and Caring Sciences, Linköping University, Linköping, Sweden

**Keywords:** Diseases, Risk factors

## Abstract

Middle-aged adults engaging in unhealthy lifestyle behaviors are at higher risk of chronic diseases. However, little is known about the co-occurrence of these behaviors and their determinants. This cohort study examined the co-occurrence of unhealthy lifestyle behaviors (alcohol consumption, diet, physical inactivity, and smoking) in 30,154 middle-aged adults and their associations with sociodemographic factors, social support, and disease history. Alcohol use was measured by the AUDIT, diet by the MiniMeal-Q, and physical inactivity and smoking by single questions. Participants had a mean age of 58 years, with 51% being female. Of them, 14% had no unhealthy behaviors, 38% had one, 36% had two, 10% had three, and 2% had all four. The most common co-occurrence was between physical inactivity and poor diet (38%). Higher education decreased the likelihood of having three or four unhealthy behaviors, while financial difficulties, having no one around who appreciated one’s efforts, and suffering of a lung disease increased it. In conclusion, middle-aged adults exhibit varying levels of unhealthy lifestyle behaviors. Higher education is linked to reduced engagement in multiple unhealthy behaviors, whereas financial strain, lower quality of social support, and lung disease increase the risk.

## Introduction

The prevalence of unhealthy lifestyle behaviours, such as excessive alcohol consumption, smoking, physical inactivity, and poor adherence to dietary recommendations, has been a growing concern in public health^1,2^. These unhealthy lifestyle behaviours can have significant negative impacts on individuals' health and increase the risk of chronic diseases^1,2^. Unhealthy lifestyle behaviours have been estimated to account for almost two-thirds of cardiovascular deaths in low-, middle- and high-income countries^3^, and approximately one-third of cancer cases could be prevented by improving lifestyle behaviours. Co-occurrence of unhealthy lifestyle behaviours reduces considerably the number of remaining years of life expected to live without disability and without chronic conditions^4^.

While studies have examined the co-occurrence of unhealthy lifestyle behaviours in the general adult population^5,6^, there is a limited understanding of the co-occurrence of multiple unhealthy behaviours in middle-aged adults. Based on studies, 15% of adults do not engage in unhealthy lifestyle behaviours^5^. In middle-aged and older adults, a higher percentage of participants avoid these behaviours, 28% and 31%, respectively^7,8^. The highest co-occurrence in the adult population was observed between physical inactivity and non-adherence to dietary recommendations (approximately 50%). Additionally, non-adherence to dietary recommendations and smoking were identified in 23–38% of the adult population^5^. One study^7^ in middle-aged adults in Australia studied the co-occurrence of six unhealthy lifestyle behaviours (smoking, unhealthy alcohol behaviour, unhealthy dietary behaviour, physical inactivity, sedentary behaviour, and insufficient sleep). This study showed that 31% of participants reported no risk behaviour, 37% had one unhealthy lifestyle behaviour, and 21%, 8%, 2%, 0.4%, and 0.04% had respectively two, three, four, five and six unhealthy lifestyle behaviours. Another Swedish study^8^ examined the co-occurrence of unhealthy lifestyle behaviours (smoking, risky alcohol consumption, insufficient physical activity, and poor diet) in middle-aged adults and found that 50% of the middle-aged adults had two unhealthy lifestyle behaviours and 18% had three.

A systematic literature review on the co-occurrence of unhealthy lifestyle behaviours in the adult population described the co-occurrence of harmful alcohol consumption and smoking as well as unhealthy diet and smoking^5^. Inconsistencies with respect to age groups were found in the literature, with one study^9^ showing that individuals aged 45–64 demonstrated lower likelihoods of engaging in two, three, or four risk behaviours compared to those aged 16–24, while another study revealed a contrasting pattern, as older participants (25–34 years, 35–44 years, 45–54 years) reported engaging in more risk behaviours than their younger counterparts (16–24 years)^10^. Socio-economic status and social support are well-known factors influencing the co-occurrence of unhealthy lifestyle behaviours. Socio-economic status is considered the strongest predictor of engaging in multiple health risk behaviours^5,11^. Additionally, good social support helps protect against adopting unhealthy lifestyle behaviours^8,12^. Overall, the literature is limited in middle-aged adults in being able to describe the prevalence of co-occurring unhealthy lifestyle behaviours, as well as how the prevalence differentiates between groups in society.

To develop targeted interventions for middle-aged adults which promote multiple lifestyle behaviours, we need to expand upon what is known about the prevalence of co-occurrence in this population. In addition, in order to tailor intervention content to specific target groups, it is necessary to estimate co-occurrence’s association with individuals’ characteristics^13^.

Therefore, this study investigated the co-occurrence of unhealthy lifestyle behaviours in a study population based of a random sample of middle-aged adults and the associations among unhealthy behaviours (alcohol consumption, diet, physical inactivity, and smoking). A secondary aim of this study was to investigate the associations between the co-occurrence of unhealthy lifestyle behaviours and individual level variables which are relatively easy to monitor and could, therefore, be used to defined groups of individuals who may benefit from, and be targets for, health behaviour interventions. These individual level variables included sociodemographic characteristics, socioeconomic factors, social support, and history of disease.

## Methods

This study used data from the Swedish CArdioPulmonary bioImage Study (SCAPIS)^14^, a Swedish nationwide population-based cohort mainly designed to research cardiovascular and chronic obstructive pulmonary diseases. All Swedish residents have a unique personal identification number, which allowed unbiased and randomized recruitment from the Swedish population register. Initial contact was made by sending out an informational brochure asking recipient to contact the study centre. If the centre was not contacted, the recipient was reminded by up to three telephone calls and finally by letter. If the centre was contacted and the recipient was willing to participate in the study, an appointment was arranged at the study centre. No exclusion criteria applied except the inability to understand written and spoken Swedish for informed consent.

A protocol of the statistical analysis plan, was registered on the open science framework before start of the analysis^15^. The Regional Ethical Review Board approved SCAPIS as a multicentre trial (2014-33-32 M). All participants provided written informed consent, and the research was conducted in accordance with the Declaration of Helsinki. This study received ethical approval on 2021-05-18 from the Swedish Ethical Review Authority (Dnr 2021-02,121).

### Outcomes and measures

Participants of SCAPIS were asked to complete a questionnaire, comprising of 140 questions, including questions on biological sex, age, socioeconomic factors, social support, history of disease, family history of disease, alcohol consumption, smoking, physical inactivity, and intake of food and drinks.

Socioeconomic factors assessed included educational level, employment status, marital status, financial preparedness for unforeseen expenses, budget management difficulties, living situation, and own and parents’ country of birth. Social support was evaluated using a validated condensed version of the Interview Schedule for Social Interaction^16^, with each of the twelve items assessed separately. Analysing each item separately allowed us to study which parts of social support specifically is associated with health behaviours, rather than only looking at social support as a whole, summarised into a single score. Self-reported medical history encompassed various conditions, including heart-related conditions (e.g. myocardial infarction and heart failure), lung diseases (e.g. chronic obstructive pulmonary disease (COPD) and chronic bronchitis), diabetes, and cancers. Family history of diseases in first-degree relatives was also queried.

*Unhealthy alcohol consumption* was assessed using the alcohol use disorders identification test (AUDIT)^17^. In this study, the last two questions (“Have you or someone else been injured because of your drinking?” and “Has a relative, friend, doctor, or other health care worker been concerned about your drinking or suggested you cut down?”) had only two response options (No/Yes) instead of the usual three (No/Yes, but not in the last year/Yes, during the last year). The AUDIT score ranges from 0 to 40, with higher scores indicating more harmful alcohol use. A score of 8 or higher typically indicates hazardous or harmful alcohol consumption. The Cronbach’s alpha in this study was 0.73.

*Nonadherence to dietary recommendations* was determined through food intake assessments using the MiniMeal-Q questionnaire^18^. This questionnaire covered 75 to 126 food items and inquired about dietary habits over the past few months. Energy (kJ/day) and macronutrient (g/day) intake were calculated using the national nutrient content database (www.slv.se). The Swedish Healthy Eating Index score (SHEI-score) was utilised to gauge adherence to dietary recommendations^19^. This index reflects the ratio between the recommended consumption of nine specified foods and the nutrient intake outlined in the 2012 Nordic Recommendations. These nine specified foods and the nutrient intake were:

1. At least 500 g vegetables and fruit per day (potatoes not included)
2. At least 2.5 g fibre/MJ
3. At least 75 g wholemeal/10 MJ
4. At least 45 g fish and shellfish per day (frequency of 2–3 per week and portion size 125 g)
5. Polyunsaturated Fatty Acids (PUFA) minimum 7.5 E%
6. Monounsaturated fatty acids (MUFA) minimum 15 E%
7. Saturated Fatty Acids (SFA) maximum 10 E%
8. Maximum 500 g red and processed meat per week
9. Maximum 10 E% added sugar

Scores for each item range from 0 (nonadherence to the dietary recommendation) to 1 (adherent to the dietary recommendation), and the total score ranges from 0 to 9, with lower scores indicating poorer adherence to dietary recommendations. It’s worth noting that, for consistency with other behaviour measures, the scale was reversed in the analyses enclosed, so higher scores indicate lower adherence and vice versa. Participants in the first, second, and third quartiles were considered non-adherent to dietary guidelines.

*Physical inactivity* was defined following the definition by the World Health Organization as insufficient physical activity to meet current recommendations^20^. For adults aged 18–64, this entails at least 150–300 min of moderate-intensity aerobic activity or at least 75–150 min of vigorous-intensity aerobic activity per week. It should also include muscle-strengthening activities involving all major muscle groups two or more days per week. Physical inactivity was assessed with a question regarding exercise frequency over the past three months to improve endurance and well-being. Response options included: Never; Sometimes, but not regularly; 1–2 times a week; 2–3 times a week; More than 3 times a week; Not willing/able to reply. “Never” or “Sometimes, but not regularly” indicated physical inactivity as these participants were considered to not have sufficient physical activity to meet current recommendations.

*Smoking status* was determined with a single question: “Do you smoke?” Response options included: No, I never smoked; No, I quit smoking; Yes, I smoke occasionally; Yes, I smoke regularly; Not willing/able to reply. Participants who stated they were current smokers during an oral inquiry at a study visit, but answered on the questionnaire that they did not smoke, were considered current smokers.

### Data analyses

We used multilevel multinomial regression to estimate the cohort prevalence of co-occurrence of two and three unhealthy lifestyle behaviours and the prevalence of all four unhealthy lifestyle behaviours. The levels were the six different study centres for which adaptive intercepts were added to the models. ‘No unhealthy behaviours’ was used as the reference category, and adaptive intercepts were added for study site. We added covariates to the multinomial regression models to estimate conditional associations between the co-occurrence of unhealthy lifestyle behaviours and sociodemographic characteristics (including age and biological sex), socioeconomic factors, social support, and history of disease. To estimate the associations among unhealthy lifestyle behaviours, we regressed each health behaviour measure (AUDIT, SHEI-score, physical inactivity, and smoking) against the other health behaviour measures, e.g., for AUDIT, we used SHEI-score, physical inactivity, and smoking as covariates. Interactions among the covariates were also added. We used negative binomial regression for AUDIT, linear regression for SHEI-scores, and logistic regression for physical inactivity and smoking. These models also included adaptive intercepts for study site.

We used Bayesian inference to estimate posterior distributions of associations^21^. Unlike maximum likelihood inference with null hypothesis testing, Bayesian inference results in a probability distribution over quantities of interest (in this case, associations). Thus, rather than focusing only on rejecting or keeping the narrow null hypothesis, Bayesian inference assigns a probability to each possible value of the associative measure (e.g. an odds ratio). This allows for a more straightforward inspection of the relative compatibility between the data and different association estimates. The distribution of estimates is referred to as the *posterior* distribution since it incorporates our prior belief (*prior* distribution) and the data observed. For reporting it is convenient to communicate a point estimate of the association rather than the full posterior distribution, and we used the median of the posterior distribution for this purpose. To portray the posterior distribution and the inherent uncertainty of the estimates, we used 95% compatibility intervals (CI) defined as the 2.5% and 97.5% percentiles of the posterior distributions. We used standard normal priors for all parameters in the multinomial cohort prevalence models. In models with covariates, we used Cauchy priors to induce sparsity, with a half-normal hyperprior for the Cauchy scale parameter. Data were analysed using the R statistical software version 4.0.4 and Stan 2.30.1 (CmdStan).

## Results

A total of 59,909 individuals were invited to participate in SCAPIS, with 30,154 (50.3%) choosing to join. On average, participants were 58 years old (SD = 4), and 51% were female (n = 15,508). The majority were married (71%, n = 21,514), possessed at least a secondary school education (88%, n = 26,553), were employed (81%, n = 24,483), and were born in Europe (91%, n = 27,519). Prevalent diseases, affecting ten percent or more of the population, included hypertension (22%, n = 6,620) and hyperlipidaemia (11%, n = 3,425). Over a quarter of participants had a family history of myocardial infarction (27%, n = 8,299), diabetes (26%, n = 7,929), and stroke (26%, n = 7,766) (Supplementary Table 1).

The median posterior AUDIT score in the cohort was 3.8 (95% CI = 3.44; 4.08), and the median posterior prevalence of hazardous and harmful alcohol consumption was 7.7% (95% CI = 6.1%; 10.8%). The median posterior Swedish Healthy Eating Index score (reversed) was 6.0 (95% CI = 5.7; 6.2). The median posterior prevalence of physical inactivity was 50% (95% CI = 46.3%; 53.5%), and the median posterior prevalence of current smokers was 12.8% (95% CI = 9.2%; 18.8%).

### Prevalence of co-occurrence of unhealthy lifestyle behaviours

**Fig. 1:**
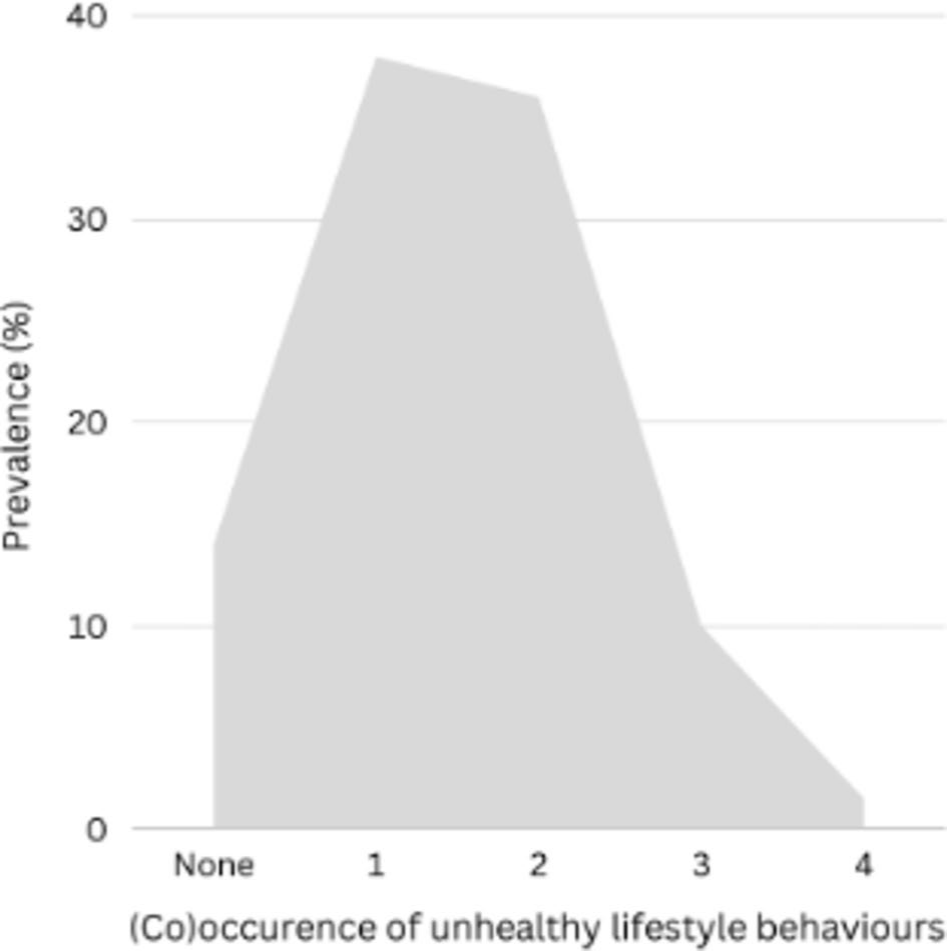
Prevalence of (co)occurrence of unhealthy lifestyle behaviours (unhealthy alcohol consumption, smoking, sedentary lifestyle and non-adherence to dietary recommendations).

Figure 1 depicts the prevalence of co-occurrence of unhealthy lifestyle behaviours. Data was not available to calculate the number of unhealthy behaviours for 17% (n = 5130) of participants; therefore, when estimating prevalences of the number of unhealthy behaviours we included participants with data available for all behaviours. In total, 14% of participants did not engage in unhealthy lifestyle behaviours (n = 3500). A single unhealthy behaviour was observed among 38% of participants (n = 9613), while two unhealthy behaviours were observed among 36% (n = 9051). Ten per cent of participants were observed to have three unhealthy behaviours (n = 2497), and only 2% were observed to have all four (n = 363).

### Prevalence and conditional associations of the co-occurrence of unhealthy lifestyle behaviours

In Supplementary Table 2, the estimated prevalence of combined unhealthy lifestyle behaviours from multilevel logistic regression are presented. The most prevalent combination of unhealthy lifestyle behaviours was physical inactivity paired with non-adherence to dietary recommendations, estimated to 38% (n = 10,573). The prevalence of smoking in conjunction with nonadherence to dietary recommendations was estimated to 9% (n = 2742), while the prevalence of smoking combined with physical inactivity was estimated to 8% (n = 2522). The prevalence of unhealthy alcohol consumption alongside nonadherence to dietary guidelines was estimated to 7% (n = 2093), and the prevalence of unhealthy alcohol consumption paired with physical inactivity was estimated to 5% (n = 1576). Finally, the prevalence of both unhealthy alcohol consumption and smoking was estimated to 2% (n = 643).

**Fig. 2:**
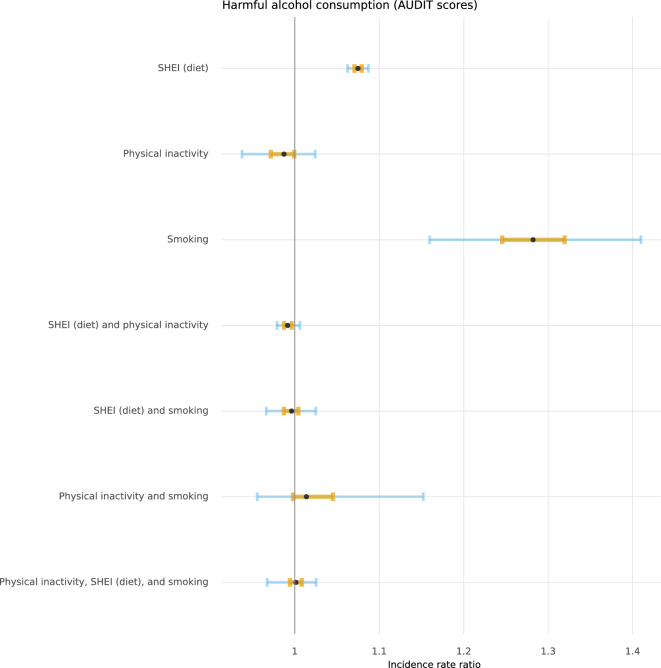
Posterior distributions of conditional associations between AUDIT scores and diet (SHEI), physical inactivity, and smoking. Points represent the median of the posterior distributions, orange bars the interquartile range, and blue bars the 2.5% and 97.5% percentiles.

**Fig. 3:**
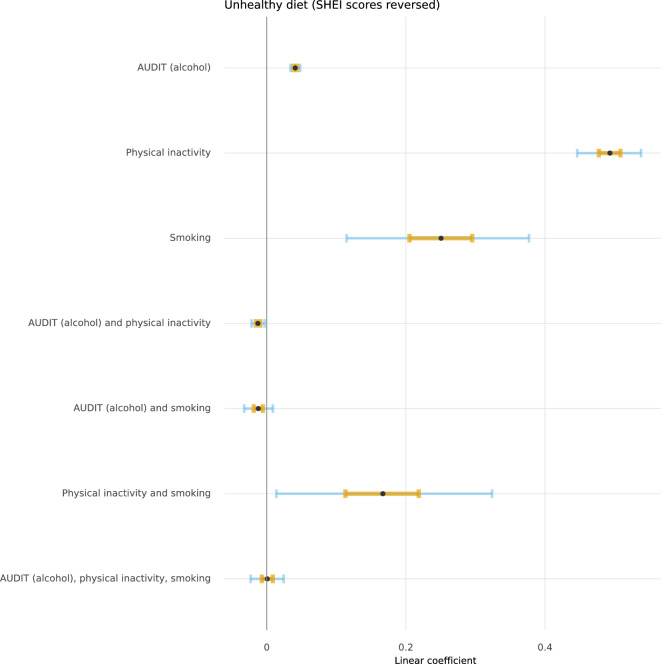
Posterior distributions of conditional associations between SHEI scores and AUDIT, physical inactivity, and smoking. Points represent the median of the posterior distributions, orange bars the interquartile range, and blue bars the 2.5% and 97.5% percentiles.

**Fig. 4:**
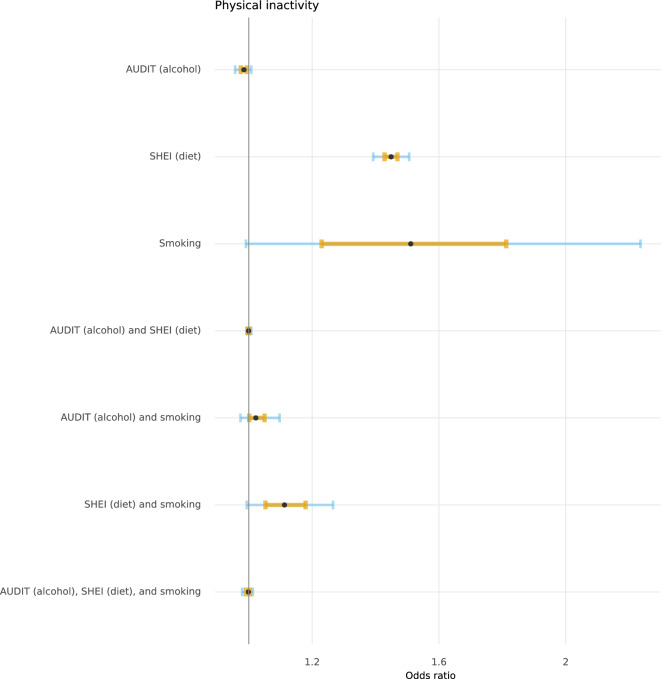
Posterior distributions of conditional associations between physical inactivity and AUDIT, SHEI, and smoking. Points represent the median of the posterior distributions, orange bars the interquartile range, and blue bars the 2.5% and 97.5% percentiles.

**Fig. 5:**
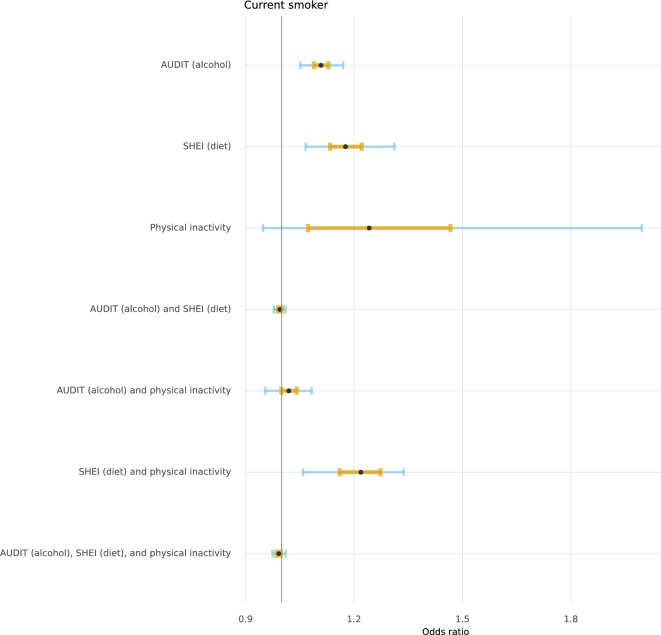
Posterior distributions of conditional associations between smoking and AUDIT, SHEI, and physical inactivity. Points represent the median of the posterior distributions, orange bars the interquartile range, and blue bars the 2.5% and 97.5% percentiles.

Figures 2, 3, 4, and 5 depict the posterior distributions of the estimated associations among unhealthy lifestyle behaviours. Participants who were smokers scored higher on AUDIT, after having adjusted for the other two lifestyle behaviours. Similarly, participants with less adherence to dietary recommendations also scored higher on AUDIT. Smoking was more likely among physically inactive participants and participants who were less adherent to dietary recommendations. The combination of both physical inactivity and non-adherence to dietary recommendations further increased the likelihood of smoking. Being less adherent to dietary recommendations was also associated with being physically inactive. Please see Supplementary Tables 3 through 6 for full numerical details.

### Factors associated with co-occurrence of unhealthy lifestyle behaviours

**Fig. 6:**
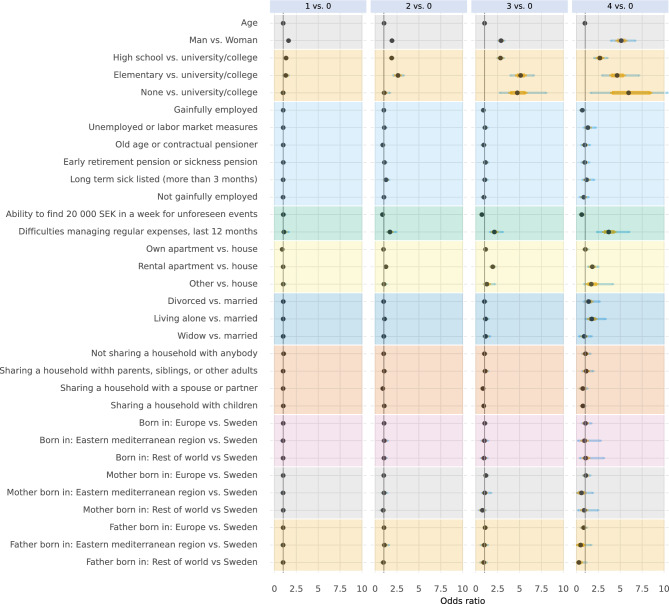
Conditional associations between socioeconomic factors and the number of unhealthy lifestyle behaviours. Associations are given in odds ratios with no unhealthy lifestyle behaviours as reference category. Points represent the median of the posterior distributions, orange bars the interquartile range, and blue bars the 2.5% and 97.5% percentiles.

**Fig. 7:**
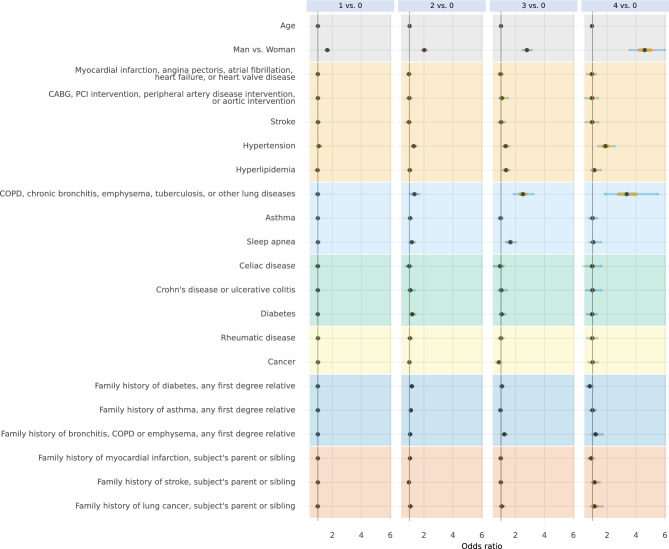
Conditional associations between social support and the number of unhealthy lifestyle behaviours. Associations are given in odds ratios with no unhealthy lifestyle behaviours as reference category. Points represent the median of the posterior distributions, orange bars the interquartile range, and blue bars the 2.5% and 97.5% percentiles.

**Fig. 8:**
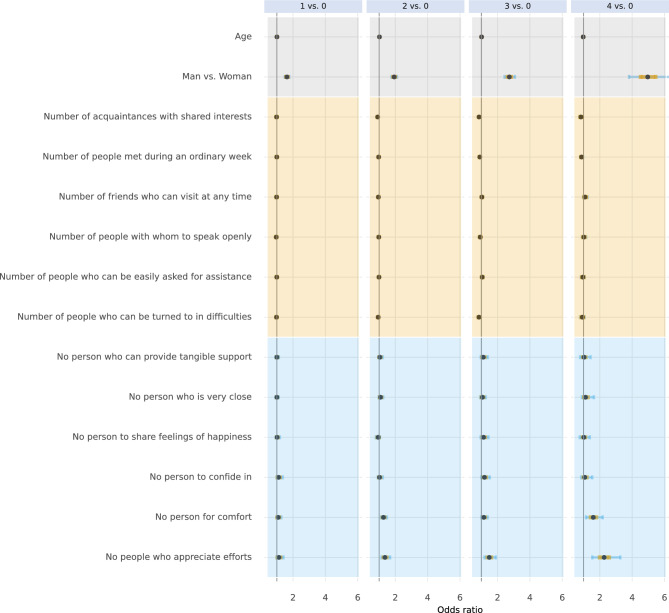
Conditional associations between history of disease and family history of disease and the number of unhealthy lifestyle behaviours. Associations are given in odds ratios with no unhealthy lifestyle behaviours as reference category. Points represent the median of the posterior distributions, orange bars the interquartile range, and blue bars the 2.5% and 97.5% percentiles.

In Figs. 6, 7, and 8, posterior distributions are presented of estimated conditional associations between the number of unhealthy lifestyle behaviours and: socioeconomic factors (Fig. 6), social support (Fig. 7), and history of disease and family history of disease (Fig. 8). For full numerical details, please see Supplementary Tables 7 to 9. Men, even when accounting for socioeconomic factors, social support, and history of disease and family history of disease, tended to have more often co-occurrence of unhealthy lifestyle behaviours compared to women. Age did not appear to play a major role in the co-occurrence of these behaviours, noting that the age-range in the study population was narrow.

We found that participants with a university or college degree were less likely to have three or four co-occurrent unhealthy lifestyle behaviours than participants with low education those who only finished elementary school, or those with a high school degree. We also found that participants who had difficulties managing regular expenses were more likely to have three or four co-occurring unhealthy lifestyle behaviours. There was also a marked association between reporting having no one around who appreciated one’s efforts and a higher number of unhealthy lifestyle behaviours. Finally, we found that participants with a lung disease (such as COPD, chronic bronchitis, emphysema, tuberculosis, or other lung diseases) were more likely to have three or four co-occurring unhealthy lifestyle behaviours.

## Discussion

Overall, 14% of middle-aged adults did not report engaging in any of the unhealthy lifestyle behaviours measured in this study, which included harmful alcohol consumption, not adhering to dietary recommendations, physical inactivity, and smoking. A similar finding of the proportion of individuals abstaining from these behaviours was noted in population-based Eurobarometer data (15%)^6^. The Eurobarometer data study examined the prevalence of five unhealthy lifestyle behaviours, specifically smoking, excessive alcohol consumption, infrequent consumption of fresh fruits, physical inactivity, and irregular dental check-ups. However, two studies involving middle-aged and older adults found twice as many individuals not engaging in unhealthy lifestyle behaviours (28 and 31%)^7,8^.

Around one-third engaged in one or two unhealthy lifestyle behaviours, respectively. This finding aligns with population-based data from Eurobarometer^6^, which observed a prevalence of 29% for a single unhealthy lifestyle behaviour and 30% for two such behaviours among participants. Similar results for engaging in one unhealthy lifestyle behaviour (37%) was found in an Australian study, but it noted that one-fifth of the participants reported two unhealthy lifestyle behaviours^7^. Another Swedish study in middle-aged adults found that a higher amount that reported two unhealthy lifestyle behaviours (50%)^8^. Finally, we found that 10% of middle-aged adults engaged in three unhealthy lifestyle behaviours, while only 2% engaged in four. These findings are in line with Australian findings, which found that 8% of participants engaged in three unhealthy lifestyle behaviours and 2% in four^7^. A Swedish study reported approximately twice the amount of middle-aged adults engaged in three unhealthy lifestyle behaviours (18%) three, and similar amount of middle-aged adults engaged in this study in four unhealthy lifestyle behaviours (3%)^8^.

In our study, the highest co-occurrence was observed between physical inactivity and non-adherence to dietary recommendations. These findings align with studies included in a literature review involving the adult population^5^, where the prevalence of co-occurrence was found to be within the range of 47% to 54% and with a Swedish study where the co-occurrence was found in 46%^8^. Non-adherence to dietary recommendations and smoking were identified in 23–38% of the adult population^5^, whereas in our study, this co-occurrence was only prevalent in 9% of the middle-aged population. Another Swedish study showed an even lower percentage for this co-occurrence (0.4%)^8^. These results are not surprising, as the prevalence of smoking has reduced drastically in Sweden over the past decade^22^. The odds of smoking were increased among those with higher AUDIT and SHEI scores and those who were physically inactive, suggesting that smokers were more likely than not to have other unhealthy lifestyle behaviours. This was confirmed in another Swedish study, where they found that most smokers (88%) reported three or four unhealthy lifestyle behaviour.

We found that participants with a lower educational level and having difficulties managing expenses were more likely to have 3 or 4 co-occurring unhealthy behaviours. These findings are corroborated by studies included in a systematic review^5^, showing that socio-economic status was the strongest predictor of engaging in multiple health risk behaviours. We also found that middle-aged adults who felt that they had no one around who appreciated their efforts were more likely to be unhealthy with respect to all four behaviours included in this study. The importance of the quality of social support for health was confirmed by a Swedish cohort study^23^, including older adults, showing that a rich social network protected participants from having multiple unhealthy lifestyle behaviours^8^.

Most studies of public health interventions have been on interventions targeting one unhealthy lifestyle behaviour, even if it is recognised that interventions targeting more than one behaviour could be more effective^24^. A scoping review showed that it is rare that interventions target all four health behaviours studied here, and of those interventions that target multiple behaviours, more than half target unhealthy diet and insufficient physical activity (the most prevalent co-occurrence found in this study)^25^. However, several ongoing trials in a range of populations will provide evidence of the benefits and drawbacks of simultaneously targeting multiple behaviours in the near future^26–28^. Our study showed that the efficacy of multi-behavioural interventions in middle-aged adults should be focussing on simultaneous targeting physical inactivity and unhealthy diet, given their high co-occurrence in this population. Future research should also assess the underlying mechanisms in middle-aged adults driving the associations observed in our study, for example why in middle-aged adults that have lower quality of social support lead to multiple unhealthy lifestyle behaviours and assess which public health policies could impact the co-occurrence of unhealthy lifestyles. Longitudinal studies are needed to establish causal relationships between sociodemographic characteristics, socioeconomic factors, social support, and history of disease on the co-occurrence of unhealthy lifestyle behaviours.

### Methodological considerations

This cross-sectional study used statistical methods that estimated conditional associations between variables. Therefore, estimates of associations should not be considered causal as we cannot rule out confounding and collider bias. In addition, the study relied on self-reported variables, which may introduce bias^29–32^. The findings are nevertheless helpful in describing the state of the study population, as this gives insights into where targeted interventions are needed. A related limitation is that the study cannot explain the underlying reasons for the observed associations. Socio-demographic disparities persist partially due to the uneven distribution of health-related behaviours, though this study cannot explain why this continues to be the case.

Another limitation of this study is that the sample is selective, consisting of those who could and chose to participate in SCAPIS, making it less representative of all middle-aged adults in Sweden. A discrepancy can, for instance, be seen when considering harmful alcohol consumption, which has been found to be much more prevalent in general population surveys. However, the estimated associations between factors are still valid if participation was not selectively conditional on them. For instance, the association between education and the co-occurrence of unhealthy behaviours is valid if it is not different in those who participated and those who did not. While this is less likely to be the case than selectiveness based on health behaviours alone, it is still a limitation to be borne in mind when interpreting findings.

## Conclusions

This study highlights the need for targeted interventions addressing multiple unhealthy lifestyle behaviours, especially among socio-economically disadvantaged individuals and those with low-quality social support. Future research and public health efforts should consider strategies to simultaneously address unhealthy lifestyle behaviours and promote a comprehensive approach to healthy ageing in middle-aged adults.

## Supplementary Information

Supplementary Information.

## Data Availability

Availability of data and materials Due to the nature of the sensitive personal data and study materials, they cannot be made freely available. However, by contacting the corresponding author or study organization (www.scapis.org), procedures for sharing data, analytic methods, and study materials for reproducing the results or replicating the procedure can be arranged following Swedish legislation.

## References

[1] Organization WH. *Action plan for the prevention and control of noncommunicable diseases in the WHO European Region: World Health Organization. Regional Office for Europe2016.*

[2] Naghavi, M. *et al.* Global, regional, and national age-sex specific mortality for 264 causes of death, 1980–2016: A systematic analysis for the Global Burden of Disease Study 2016. *The Lancet.* **390**(10100), 1151–1210 (2017). 10.1016/S0140-6736(17)32152-9 PMC5605883 28919116

[3] Lopez, A. D., Mathers, C. D., Ezzati, M., Jamison, D. T. & Murray, C. J. Global and regional burden of disease and risk factors, 2001: systematic analysis of population health data. *Lancet* **367**(9524), 1747–1757 (2006). 10.1016/S0140-6736(06)68770-9 16731270

[4] Zaninotto, P., Head, J. & Steptoe, A. Behavioural risk factors and healthy life expectancy: Evidence from two longitudinal studies of ageing in England and the US. *Sci. Rep.* **10**(1), 6955 (2020). 32332825 10.1038/s41598-020-63843-6 PMC7181761

[5] Meader, N. *et al.* A systematic review on the clustering and co-occurrence of multiple risk behaviours. *BMC Public Health.* **16**(1), 1–9 (2016). 27473458 10.1186/s12889-016-3373-6 PMC4966774

[6] Kino, S., Bernabé, E. & Sabbah, W. The role of healthcare and education systems in co-occurrence of health risk behaviours in 27 European countries. *Eur. J. Public Health.* **28**(1), 186–192 (2018). 29346661 10.1093/eurpub/ckx071

[7] Ding, D., Rogers, K., van der Ploeg, H., Stamatakis, E. & Bauman, A. E. Traditional and emerging lifestyle risk behaviors and all-cause mortality in middle-aged and older adults: Evidence from a large population-based Australian cohort. *PLoS Med.* **12**(12), e1001917 (2015). 26645683 10.1371/journal.pmed.1001917 PMC4672919

[8] Thomas, K. *et al.* Associations of psychosocial factors with multiple health behaviors: A population-based study of middle-aged men and women. *Int. J. Environ. Res. Public Health.* **17**(4), 1239 (2020). 32075162 10.3390/ijerph17041239 PMC7068361

[9] Poortinga, W. The prevalence and clustering of four major lifestyle risk factors in an English adult population. *Prev. Med.* **44**(2), 124–128 (2007). 17157369 10.1016/j.ypmed.2006.10.006

[10] Lawder, R. *et al.* Is the Scottish population living dangerously? Prevalence of multiple risk factors: the Scottish Health Survey 2003. *BMC Public Health.* **10**(1), 1–13 (2010). 20540711 10.1186/1471-2458-10-330 PMC2903518

[11] Foster, H. M. *et al.* The effect of socioeconomic deprivation on the association between an extended measurement of unhealthy lifestyle factors and health outcomes: A prospective analysis of the UK Biobank cohort. *The Lancet Public Health.* **3**(12), e576–e585 (2018). 30467019 10.1016/S2468-2667(18)30200-7

[12] Ortiz, C. *et al.* Physical and social environmental factors related to co-occurrence of unhealthy lifestyle behaviors. *Health Place.* **75**, 102804 (2022). 35462183 10.1016/j.healthplace.2022.102804

[13] McAloney, K., Graham, H., Law, C. & Platt, L. A scoping review of statistical approaches to the analysis of multiple health-related behaviours. *Prev. Med.* **56**(6), 365–371 (2013). 23518213 10.1016/j.ypmed.2013.03.002

[14] Bergström, G. *et al.* The Swedish cardiopulmonary BioImage study: objectives and design. *J. Intern. Med.* **278**(6), 645–659 (2015). 26096600 10.1111/joim.12384 PMC4744991

[15] Bendtsen BK, L. Characterising lifestyle behaviours and their associations with prevalence and risk factors of diabetes and cardiovascular disease among participants in the SCAPIS study. OSF Registries. 2023. https://osf.io/mafkd. Accessed 25-04-2024 2023.

[16] Undén, A.-L. & Orth-Gomér, K. Development of a social support instrument for use in population surveys. *Soc Sci. Med.* **29**(12), 1387–1392 (1989). 2629121 10.1016/0277-9536(89)90240-2

[17] Saunders, J. B., Aasland, O. G., Babor, T. F., De la Fuente, J. R. & Grant, M. Development of the alcohol use disorders identification test (AUDIT): WHO collaborative project on early detection of persons with harmful alcohol consumption-II. *Addiction.* **88**(6), 791–804 (1993). 8329970 10.1111/j.1360-0443.1993.tb02093.x

[18] Christensen, S. E. *et al.* Two new meal-and web-based interactive food frequency questionnaires: Validation of energy and macronutrient intake. *J. Med. Internet Res.* **15**(6), e2458 (2013). 10.2196/jmir.2458 PMC3713929 23739995

[19] Moraeus, L., Lindroos, A. K., Lemming, E. W. & Mattisson, I. Diet diversity score and healthy eating index in relation to diet quality and socio-demographic factors: Results from a cross-sectional national dietary survey of Swedish adolescents. *Public Health Nutr.* **23**(10), 1754–1765 (2020). 32301415 10.1017/S1368980019004671 PMC7267781

[20] WHO guidelines on physical activity and sedentary behaviour: at a glance. https://apps.who.int/iris/bitstream/handle/10665/337001/9789240014886-eng.pdf. 2020. https://apps.who.int/iris/bitstream/handle/10665/337001/9789240014886-eng.pdf. Accessed 05–07 2023.

[21] Bendtsen, M. A gentle introduction to the comparison between null hypothesis testing and Bayesian analysis: reanalysis of two randomized controlled trials. *J. Med. Internet Res.* **20**(10), e10873 (2018). 30148453 10.2196/10873 PMC6231868

[22] Borna, E. *et al.* Changes in the prevalence of asthma and respiratory symptoms in western Sweden between 2008 and 2016. *Allergy.* **74**(9), 1703–1715 (2019). 31021427 10.1111/all.13840

[23] Rizzuto, D., Orsini, N., Qiu, C., Wang, H.-X., Fratiglioni, L. Lifestyle, social factors, and survival after age 75: Population based study. *BMJ*.;345 (2012). 10.1136/bmj.e5568 PMC3431442 22936786

[24] Prochaska, J. J. & Prochaska, J. O. A review of multiple health behavior change interventions for primary prevention. *Am. J. Lifestyle Med.* **5**(3), 208–221 (2011). 10.1177/1559827610391883 PMC3865280 24358034

[25] King, K. *et al.* Characteristics of interventions targeting multiple lifestyle risk behaviours in adult populations: A systematic scoping review. *PloS One.* **10**(1), e0117015 (2015). 25617783 10.1371/journal.pone.0117015 PMC4305300

[26] Bendtsen, M. *et al.* The Mobile health multiple lifestyle behavior interventions across the lifespan (MoBILE) research program: Protocol for development, evaluation, and implementation. *JMIR Res. Protoc.* **9**(4), e14894 (2020). 32310147 10.2196/14894 PMC7199135

[27] Åsberg, K. *et al.* Digital multiple health behaviour change intervention targeting online help seekers: Protocol for the COACH randomised factorial trial. *BMJ Open.* **12**(7), e061024 (2022). 35882466 10.1136/bmjopen-2022-061024 PMC9330315

[28] Åsberg, K. *et al.* Multiple lifestyle behaviour mHealth intervention targeting Swedish college and university students: Protocol for the Buddy randomised factorial trial. *BMJ Open.* **11**(12), e051044 (2021).

[29] Livingston, M. & Callinan, S. Underreporting in alcohol surveys: Whose drinking is underestimated?. *J. Stud. Alcohol Drugs* **76**(1), 158–164 (2015). 25486405

[30] Gallus, S. *et al.* Temporal changes of under-reporting of cigarette consumption in population-based studies. *Tob. Control.* **20**(1), 34–39 (2011). 20861005 10.1136/tc.2009.034132

[31] Prince, S. A. *et al.* A comparison of direct versus self-report measures for assessing physical activity in adults: A systematic review. *Int. J. Behav. Nutr. Phys. Act.* **5**(1), 1–24 (2008). 18990237 10.1186/1479-5868-5-56 PMC2588639

[32] Ravelli, M. N. & Schoeller, D. A. Traditional self-reported dietary instruments are prone to inaccuracies and new approaches are needed. *Front. Nutr.* **7**, 90 (2020). 32719809 10.3389/fnut.2020.00090 PMC7350526

