# Supplementary material for: Co-occurrence of unhealthy lifestyle behaviours in middle-aged adults: findings from the Swedish CArdioPulmonary bioImage Study (SCAPIS)
Source: Sci Rep. 2024 Oct 1;14:22853. doi: 10.1038/s41598-024-71092-0 (PMC11445527; doi:10.1038/s41598-024-71092-0)
Supplement: Supplementary file 1 — Supplementary Information. [file 41598_2024_71092_MOESM1_ESM.docx]

Co-occurrence of unhealthy lifestyle behaviours in middle-aged adults: findings from the Swedish CArdioPulmonary bioImage Study (SCAPIS)

Supplementary tables

Supplementary Table 1 - Demographics, socio-economic factors, social interaction, social support, self-reported history of disease and family history of disease of 30154 participants aged 50 to 65

| **Age (mean, sd)** | 58 (4) |
| --- | --- |
| **Female gender (%, n)** | 51% (15508) |
| **Education (%, n)** |  |
| No education | 0.7% (197) |
| Primary school | 9% (2550) |
| Secondary school | 44% (13335) |
| University | 44% (13218) |
| **Employment status (%, n)** |  |
| Gainfully Employed | 81% (24483) |
| Early retirement or sickness pension | 6% (1654) |
| Old age or contractual pensioner | 5% (1535) |
| Unemployed or labour market measures | 4% (1072) |
| Other not gainfully employed (including leave of absence, parental leave, studying or training) | 3% (829) |
| Long term sick listed (more than 3 months) | 2% (681) |
| **Ability to find 1 800 EUR in a week (%, n)** | 88% (26540) |
| **Difficulties managing expenses (%, n)** | 5% (1608) |
| **Type of residence (%, n)** |  |
| Own house | 45% (13677) |
| Own apartment | 29% (8712) |
| Rental Apartment | 22% (6691) |
| **Marital Status (%, n)** |  |
| Married | 71% (21514) |
| Alone | 13% (3975) |
| Divorced | 11% (3275) |
| Widow | 2% (491) |
| **Living situation (%, n)** |  |
| With spouse or partner | 71% (21403) |
| With children | 28% (8281) |
| Alone | 19% (5651) |
| With parents, sibling, or other adults | 2% (719) |
| **Region of birth (%, n)** |  |
| European | 91% (27519) |
| Eastern Mediterranean | 3% (1017) |
| Americans | 1% (401) |
| African/Southeast Asian/Western Pacific | 1% (373) |
| **Region of birth father (%, n)** |  |
| European | 87% (26077) |
| Eastern Mediterranean | 3% (948) |
| Americans | 1% (411) |
| African/Southeast Asian/Western Pacific | 1% (372) |
| **Region of birth mother (%, n)** |  |
| European | 87% (26155) |
| Eastern Mediterranean | 3% (941) |
| Americans | 1% (394) |
| African/Southeast Asian/Western Pacific | 1% (358) |
| Missing | 8% (2306) |
| **Social interaction** |  |
| Number acquaintances with shared interests (mean, sd) | 3 (1) |
| Number people met during an ordinary week (mean, sd) | 3 (1) |
| Number people who can easily be asked for assistance (mean, sd) | 3 (1) |
| Number of friends that you can visit any time (mean, sd) | 2 (1) |
| Number of people with whom speak openly (mean, sd) | 2 (1) |
| Number of people who to turn to in difficulties (mean, sd) | 2 (1) |
| **Social support (%, n)** |  |
| Tangible support | 89% (26843) |
| Person to share feelings of happiness | 88% (26561) |
| Person who is close | 86% (25869) |
| Person to confide | 84% (25365) |
| Person who appreciates your efforts | 84% (25459) |
| Person to comfort | 75% (22523) |
| **Self-reported history of disease (%, n)** |  |
| Hypertension | 22% (6620) |
| Hyperlipidemia | 11% (3425) |
| Asthma | 8% (2422) |
| Cancer | 6% (1738) |
| Myocardial infarction, angina pectoris, atrial fibrillation, heart failure, or heart valve | 5% (1436) |
| Sleep apnea | 4% (1292) |
| Diabetes | 4% (1291) |
| Rheumatic disease | 4% (1083) |
| Chronic obstructive pulmonary disease, chronic bronchitis, emphysema, tuberculosis, or other lung diseases | 3% (804) |
| CABG, PCI intervention, peripheral artery disease intervention, or aortic intervention | 1% (428) |
| Stroke | 1% (421) |
| Crohn's disease or ulcerative colitis | 1% (325) |
| Celiac disease | 0.6% (186) |
| **Self-reported family history of disease (%, n)** |  |
| Myocardial infarction | 27% (8199) |
| Diabetes | 26% (7929) |
| Stroke | 26% (7766) |
| Asthma | 21% (6365) |
| Bronchitis, Chronic obstructive pulmonary disease or emphysema | 15% (4465) |
| Lung cancer | 7% (2032) |

Supplementary Table 2 - The estimated prevalence and count of each combination of unhealthy lifestyle behaviours from multilevel logistic regression.

|  | **Unhealthy alcohol consumption** | **Smoking** | **Physical inactivity** | **Non-adherence to dietary recommendations** |
| --- | --- | --- | --- | --- |
|  | **Est. (95% CI) (n)** | **Est. (95% CI) (n)** | **Est. (95% CI) (n)** | **Est. (95% CI) (n)** |
| **Smoking** | 2%  (2%;7%)  (n = 643) | -- | 8%  (6%;13%)  (n = 2522) | 9%  (7%;14%)  (n = 2742) |
| **Physical inactivity** | 5%  (4%;7%)  (n = 1576) | 8%  (6%;13%)  (n = 2522) | - | 38%  (35%;42%)  (n = 10,573) |
| **Non-adherence to dietary recommendations** | 7%  (6%;10%)  (n = 2093) | 9%  (7%;14%)  (n = 2742) | 38%  (35%;42%)  (n = 10,573) | - |

Supplementary Table 3 – Conditional associations (incidence rate ratios) between AUDIT scores and SHEI, physical inactivity, and smoking.

|  | **Posterior distribution over incidence rate ratios** | |
| --- | --- | --- |
|  | **Median (95% CI)^a^** | **Post. Prob >/< null^b^** |
| SHEI (diet) | 1.07 (1.06; 1.09) | > 99.9% |
| Physical inactivity | 0.99 (0.94; 1.02) | 76.0% |
| Smoking | 1.28 (1.16; 1.41) | > 99.9% |
| SHEI (diet) and physical inactivity | 0.99 (0.98; 1.01) | 87.4% |
| SHEI (diet) and smoking | 1.0 (0.97; 1.02) | 62.7% |
| Physical inactivity and smoking | 1.01 (0.96; 1.15) | 72.0% |
| Physical inactivity, SHEI (diet), and smoking | 1.0 (0.97; 1.03) | 55.9% |
| ^a^ Median of the posterior distribution with 95% compatibility intervals (CI) defined by 2.5% and 97.5% percentiles of the posterior distribution.  ^b^ The proportion of the posterior distribution that is above or below the null in the direction of the median. | | |

Supplementary Table 4 – Conditional associations (mean differences) between SHEI scores and AUDIT, physical inactivity, and smoking.

|  | **Posterior distribution over mean difference** | |
| --- | --- | --- |
|  | **Median (95 CI)** | **Post. prob >/< null** |
| AUDIT (alcohol) | 0.04 (0.03; 0.05) | > 99.9% |
| Physical inactivity | 0.49 (0.45; 0.54) | > 99.9% |
| Smoking | 0.25 (0.11; 0.38) | > 99.9% |
| AUDIT (alcohol) and physical inactivity | -0.01 (-0.02; -0.0) | 99.6% |
| AUDIT (alcohol) and smoking | -0.01 (-0.03; 0.01) | 88.8% |
| Physical inactivity and smoking | 0.17 (0.01; 0.32) | 98.8% |
| AUDIT (alcohol), physical inactivity, smoking | 0.0 (-0.02; 0.02) | 52.7% |
| ^a^ Median of the posterior distribution with 95% compatibility intervals (CI) defined by 2.5% and 97.5% percentiles. ^b^ The proportion of the posterior distribution that is above or below the null in the direction of the median. | | |

Supplementary Table 5 – Conditional associations (odds ratios) between physical inactivity and AUDIT, SHEI, and smoking.

|  | **Posterior distribution over odds ratios** | |
| --- | --- | --- |
|  | **Median (95 CI)** | **Post. Prob >/< null** |
| AUDIT (alcohol) | 0.98 (0.96; 1.01) | 89.7% |
| SHEI (diet) | 1.45 (1.39; 1.51) | > 99.9% |
| Smoking | 1.51 (0.99; 2.24) | 96.4% |
| AUDIT (alcohol) and SHEI (diet) | 1.0 (0.99; 1.01) | 55.6% |
| AUDIT (alcohol) and smoking | 1.02 (0.97; 1.1) | 76.9% |
| SHEI (diet) and smoking | 1.11 (0.99; 1.27) | 95.6% |
| AUDIT (alcohol), SHEI (diet), and smoking | 1.0 (0.98; 1.01) | 55.6% |
| ^a^ Median of the posterior distribution with 95% compatibility intervals (CI) defined by 2.5% and 97.5% percentiles ^b^ The proportion of the posterior distribution that is above or below the null in the direction of the median. | | |

Supplementary Table 6 - Conditional associations (odds ratios) between smoking and AUDIT, SHEI, and physical inactivity.

|  | **Posterior distribution over odds ratios** | |
| --- | --- | --- |
|  | **Median (95 CI)** | **Post. prob >/< null** |
| AUDIT (alcohol) | 1.11 (1.05; 1.17) | > 99.9% |
| SHEI (diet) | 1.18 (1.07; 1.31) | > 99.9% |
| Physical inactivity | 1.24 (0.95; 2.0) | 91.0% |
| AUDIT (alcohol) and SHEI (diet) | 0.99 (0.98; 1.01) | 74.0% |
| AUDIT (alcohol) and physical inactivity | 1.02 (0.95; 1.08) | 73.4% |
| SHEI (diet) and physical inactivity | 1.22 (1.06; 1.34) | 99.8% |
| AUDIT (alcohol), SHEI (diet), and physical inactivity | 0.99 (0.97; 1.01) | 81.8% |
| ^a^ Median of the posterior distribution with 95% compatibility intervals (CI) defined by 2.5% and 97.5% percentiles. ^b^ The proportion of the posterior distribution that is above or below the null in the direction of the median. | | |

Supplementary Table 7 – Conditional associations (odds ratios) between number of unhealthy lifestyle behaviours and sociodemographic and socioeconomic variables (with no unhealthy behaviours used as reference category).

| **Number of unhealthy lifestyle behaviours** | | | | | | | |
| --- | --- | --- | --- | --- | --- | --- | --- |
| **1 vs. 0** | | **2 vs. 0** | | **3 vs. 0** | | **4 vs. 0** | |
| **Median  (95 CI)^a^** | **Post. ^b^** | **Median  (95 CI)^a^** | **Post.^b^** | **Median  (95 CI)^a^** | **Post.^b^** | **Median  (95 CI)^a^** | **Post.^b^** |
| **Age** | | | | | | | |
| 1.0  (0.99; 1.01) | 67.9% | 1.02  (1.01; 1.03) | > 99.9% | 1.0  (0.99; 1.02) | 72.8% | 0.96  (0.93; 0.99) | 99.3% |
| **Man vs. Woman** | | | | | | | |
| 1.61  (1.48; 1.76) | > 99.9% | 1.93  (1.77; 2.1) | > 99.9% | 2.89  (2.57; 3.25) | > 99.9% | 5.12  (3.94; 6.72) | > 99.9% |
| **High school vs. university/college** | | | | | | | |
| 1.33  (1.22; 1.46) | > 99.9% | 1.9  (1.73; 2.08) | > 99.9% | 2.82  (2.48; 3.2) | > 99.9% | 2.67  (2.03; 3.54) | > 99.9% |
| **Elementary vs. university/college** | | | | | | | |
| 1.3  (1.0; 1.65) | 97.7% | 2.62  (2.07; 3.27) | > 99.9% | 5.13  (3.96; 6.62) | > 99.9% | 4.64  (2.94; 7.16) | > 99.9% |
| **None vs. university/college** | | | | | | | |
| 1.0  (0.79; 1.16) | 54.7% | 1.05  (0.86; 1.7) | 69.9% | 4.75  (2.75; 8.04) | > 99.9% | 5.95  (1.63;15.35) | 99.8% |
| **Gainfully employed** | | | | | | | |
| 1.0  (0.93; 1.09) | 53.2% | 1.0  (0.9; 1.12) | 52.6% | 0.87  (0.71; 1.03) | 94.4% | 0.67  (0.42; 1.0) | 97.5% |
| **Unemployed or labour market measures** | | | | | | | |
| 1.0  (0.91; 1.13) | 54.0% | 1.05  (0.93; 1.28) | 77.7% | 1.06  (0.86; 1.35) | 72.0% | 1.3  (0.83; 2.22) | 87.6% |
| **Old age or contractual pensioner** | | | | | | | |
| 1.0  (0.92; 1.13) | 58.0% | 0.88  (0.74; 1.02) | 94.9% | 0.91  (0.69; 1.11) | 80.4% | 0.95  (0.57; 1.54) | 59.3% |
| **Early retirement pension or sickness pension** | | | | | | | |
| 1.01  (0.94; 1.19) | 65.0% | 1.06  (0.95; 1.29) | 84.4% | 1.1  (0.91; 1.41) | 83.5% | 0.95  (0.58; 1.46) | 59.8% |
| **Not gainfully employed** | | | | | | | |
| 0.99  (0.84; 1.06) | 67.0% | 1.01  (0.88; 1.17) | 56.8% | 0.93  (0.7; 1.14) | 77.0% | 0.84  (0.39; 1.38) | 75.2% |
| **Ability to find 20 000 SEK in a week for unforeseen events** | | | | | | | |
| 1.01  (0.94; 1.22) | 65.4% | 0.85  (0.71; 1.0) | 97.1% | 0.7  (0.56; 0.88) | > 99.9% | 0.6  (0.39; 0.91) | 99.3% |
| **Difficulties managing regular expenses, last 12 months** | | | | | | | |
| 1.09  (0.97; 1.63) | 84.4% | 1.7  (1.36; 2.4) | > 99.9% | 2.12  (1.63; 3.09) | > 99.9% | 3.68  (2.35; 6.03) | > 99.9% |
| **Own apartment vs. house** | | | | | | | |
| 0.89  (0.8; 0.99) | 99.2% | 0.96  (0.87; 1.04) | 82.7% | 1.12  (0.99; 1.28) | 96.3% | 1.01  (0.76; 1.35) | 53.7% |
| **Rental apartment vs. house** | | | | | | | |
| 1.0  (0.91; 1.07) | 55.9% | 1.24  (1.11; 1.36) | > 99.9% | 1.94  (1.69; 2.23) | > 99.9% | 1.81  (1.32; 2.51) | > 99.9% |
| **Other vs. house** | | | | | | | |
| 1.01  (0.89; 1.25) | 56.9% | 1.01  (0.84; 1.3) | 56.4% | 1.26  (0.9; 2.2) | 89.2% | 1.69  (0.86; 4.17) | 92.2% |
| **Divorced vs. married** | | | | | | | |
| 1.01  (0.95; 1.13) | 65.6% | 0.98  (0.84; 1.09) | 67.2% | 0.99  (0.81; 1.22) | 53.6% | 1.38  (0.85; 2.65) | 89.2% |
| **Living alone vs. married** | | | | | | | |
| 1.0  (0.92; 1.1) | 52.4% | 1.07  (0.95; 1.27) | 87.7% | 1.12  (0.92; 1.43) | 86.7% | 1.75  (1.03; 3.32) | 98.2% |
| **Widow vs. married** | | | | | | | |
| 1.0  (0.88; 1.13) | 50.6% | 0.99  (0.83; 1.18) | 53.2% | 1.12  (0.88; 1.63) | 81.0% | 0.89  (0.31; 1.72) | 65.6% |
| **Not sharing a household with anybody** | | | | | | | |
| 1.05  (0.98; 1.23) | 87.0% | 0.99  (0.86; 1.11) | 60.9% | 0.99  (0.82; 1.2) | 52.3% | 1.03  (0.68; 1.59) | 57.0% |
| **Sharing a household with parents, siblings, or other adults** | | | | | | | |
| 0.99  (0.86; 1.09) | 58.9% | 1.04  (0.91; 1.25) | 73.8% | 1.07  (0.87; 1.41) | 73.6% | 1.11  (0.68; 1.92) | 68.8% |
| **Sharing a household with a spouse or partner** | | | | | | | |
| 0.99  (0.9; 1.08) | 59.2% | 0.88  (0.74; 1.01) | 96.4% | 0.82  (0.64; 1.02) | 95.8% | 0.73  (0.39; 1.24) | 86.9% |
| **Sharing a household with children** | | | | | | | |
| 1.01  (0.96; 1.1) | 70.5% | 1.05  (0.98; 1.14) | 90.0% | 0.91  (0.8; 1.03) | 93.7% | 0.73  (0.52; 0.99) | 97.8% |
| **Born in: Europe vs. Sweden** | | | | | | | |
| 1.0  (0.94; 1.12) | 57.5% | 1.03  (0.92; 1.18) | 69.6% | 1.03  (0.86; 1.25) | 62.1% | 1.03  (0.65; 1.7) | 55.6% |
| **Born in: Eastern mediterranean region vs. Sweden** | | | | | | | |
| 0.99  (0.76; 1.08) | 65.8% | 1.04  (0.85; 1.45) | 68.9% | 0.98  (0.65; 1.4) | 55.6% | 0.93  (0.18; 2.76) | 58.4% |
| **Born in: Rest of world vs Sweden** | | | | | | | |
| 0.99  (0.85; 1.1) | 57.9% | 1.02  (0.85; 1.33) | 59.8% | 0.94  (0.6; 1.32) | 65.4% | 1.04  (0.39; 3.14) | 54.8% |
| **Mother born in: Europe vs. Sweden** | | | | | | | |
| 1.0  (0.92; 1.07) | 53.4% | 0.99  (0.9; 1.09) | 55.4% | 1.15  (0.98; 1.39) | 95.0% | 1.07  (0.75; 1.58) | 66.1% |
| **Mother born in: Eastern mediterranean region vs. Sweden** | | | | | | | |
| 0.99  (0.8; 1.11) | 62.3% | 1.02  (0.79; 1.38) | 60.9% | 1.03  (0.72; 1.75) | 58.1% | 0.57  (0.03; 1.87) | 79.7% |
| **Mother born in: Rest of world vs Sweden** | | | | | | | |
| 1.0  (0.89; 1.17) | 54.1% | 0.93  (0.65; 1.09) | 80.9% | 0.76  (0.39; 1.13) | 88.3% | 0.89  (0.18; 2.48) | 62.9% |
| **Father born in: Europe vs. Sweden** | | | | | | | |
| 1.0  (0.92; 1.07) | 54.5% | 1.02  (0.92; 1.14) | 66.4% | 1.07  (0.91; 1.29) | 79.6% | 0.83  (0.5; 1.2) | 84.4% |
| **Father born in: Eastern mediterranean region vs. Sweden** | | | | | | | |
| 0.99  (0.8; 1.11) | 60.4% | 1.07  (0.89; 1.58) | 76.3% | 0.97  (0.58; 1.37) | 58.6% | 0.48  (0.03; 1.68) | 83.0% |
| **Father born in: Rest of world vs Sweden** | | | | | | | |
| 0.99  (0.84; 1.1) | 57.4% | 0.95  (0.7; 1.12) | 73.5% | 0.91  (0.54; 1.27) | 72.6% | 0.28  (0.03; 1.12) | 94.8% |
| ^a^ Median of the posterior distribution with 95% compatibility intervals (CI) defined by 2.5% and 97.5% percentiles of the posterior distribution.  ^b^ The proportion of the posterior distribution that is above or below the null in the direction of the median. | | | | | | | |

Supplementary Table 8 – Conditional associations (odds ratios) between number of unhealthy lifestyle behaviours and social support (with no unhealthy behaviours used as reference category).

| **Number of unhealthy lifestyle behaviours** | | | | | | | |
| --- | --- | --- | --- | --- | --- | --- | --- |
| **1 vs. 0** | | **2 vs. 0** | | **3 vs. 0** | | **4 vs. 0** | |
| **Median  (95 CI)^a^** | **Post.^b^** | **Median  (95 CI)^a^** | **Post.^b^** | **Median  (95 CI)^a^** | **Post.^b^** | **Median  (95 CI)^a^** | **Post.^b^** |
| **Age** | | | | | | | |
| 1.0  (1.0; 1.01) | 85.2% | 1.02  (1.01; 1.03) | > 99.9% | 1.01  (1.0; 1.02) | 96.2% | 0.97  (0.95; 1.0) | 98.0% |
| **Man vs. Woman** | | | | | | | |
| 1.62  (1.49; 1.77) | > 99.9% | 1.93  (1.77; 2.1) | > 99.9% | 2.73  (2.43; 3.07) | > 99.9% | 4.96  (3.81; 6.46) | > 99.9% |
| **Number of acquaintances with shared interests** | | | | | | | |
| 0.98  (0.95; 1.01) | 86.9% | 0.91  (0.88; 0.94) | > 99.9% | 0.86  (0.81; 0.9) | > 99.9% | 0.83  (0.74; 0.93) | > 99.9% |
| **Number of people met during an ordinary week** | | | | | | | |
| 1.0  (0.97; 1.02) | 64.0% | 0.97  (0.94; 1.0) | 96.8% | 0.9  (0.86; 0.95) | > 99.9% | 0.87  (0.79; 0.96) | 99.7% |
| **Number of friends who can visit at any time** | | | | | | | |
| 0.98  (0.94; 1.02) | 82.5% | 0.95  (0.91; 0.99) | 98.6% | 1.03  (0.97; 1.09) | 83.8% | 1.09  (0.97; 1.25) | 90.8% |
| **Number of people with whom to speak openly** | | | | | | | |
| 0.96  (0.91; 1.0) | 96.6% | 0.98  (0.93; 1.02) | 82.2% | 0.93  (0.86; 0.99) | 98.6% | 1.02  (0.9; 1.16) | 59.5% |
| **Number of people who can be easily asked for assistance** | | | | | | | |
| 0.99  (0.96; 1.03) | 63.3% | 0.99  (0.95; 1.03) | 68.2% | 1.05  (0.99; 1.12) | 93.8% | 0.95  (0.83; 1.07) | 78.7% |
| **Number of people who can be turned to in difficulties** | | | | | | | |
| 0.98  (0.92; 1.02) | 85.8% | 0.94  (0.89; 0.99) | 98.9% | 0.85  (0.78; 0.92) | > 99.9% | 0.92  (0.78; 1.06) | 88.1% |
| **No person who can provide tangible support** | | | | | | | |
| 1.0  (0.92; 1.12) | 53.9% | 1.05  (0.95; 1.22) | 81.2% | 1.12  (0.96; 1.39) | 91.5% | 1.03  (0.79; 1.43) | 60.0% |
| **No person who is very close** | | | | | | | |
| 1.0  (0.92; 1.11) | 53.0% | 1.08  (0.98; 1.25) | 93.4% | 1.06  (0.92; 1.27) | 80.2% | 1.12  (0.89; 1.63) | 81.7% |
| **No person to share feelings of happiness** | | | | | | | |
| 1.01  (0.94; 1.19) | 67.2% | 0.94  (0.8; 1.05) | 83.9% | 1.14  (0.96; 1.44) | 91.4% | 1.01  (0.76; 1.38) | 55.3% |
| **No person to confide in** | | | | | | | |
| 1.11  (0.99; 1.36) | 94.1% | 1.02  (0.92; 1.23) | 67.2% | 1.19  (0.99; 1.52) | 96.8% | 1.07  (0.85; 1.53) | 72.5% |
| **No person for comfort** | | | | | | | |
| 1.08  (0.99; 1.28) | 92.6% | 1.27  (1.13; 1.48) | > 99.9% | 1.15  (1.0; 1.38) | 97.3% | 1.6  (1.15; 2.18) | 99.8% |
| **No people who appreciate efforts** | | | | | | | |
| 1.13  (0.98; 1.41) | 92.8% | 1.36  (1.15; 1.68) | > 99.9% | 1.49  (1.2; 1.89) | > 99.9% | 2.27  (1.53; 3.27) | > 99.9% |
| ^a^ Median of the posterior distribution with 95% compatibility intervals (CI) defined by 2.5% and 97.5% percentiles of the posterior distribution.  ^b^ The proportion of the posterior distribution that is above or below the null in the direction of the median. | | | | | | | |

Supplementary Table 9 - Conditional associations (odds ratios) between number of unhealthy lifestyle behaviours and history of disease (with no unhealthy behaviours used as reference category)

| **Number of unhealthy lifestyle behaviours** | | | | | | | |
| --- | --- | --- | --- | --- | --- | --- | --- |
| **1 vs. 0** | | **2 vs. 0** | | **3 vs. 0** | | **4 vs. 0** | |
| **Median  (95 CI)^a^** | **Post.^b^** | **Median  (95 CI)^a^** | **Post.^b^** | **Median  (95 CI)^a^** | **Post.^b^** | **Median  (95 CI)^a^** | **Post.^b^** |
| **Age** | | | | | | | |
| 1.0  (0.99; 1.01) | 70.8% | 1.01  (1.0; 1.02) | 99.8% | 1.0  (0.99; 1.01) | 54.1% | 0.96  (0.93; 0.99) | 99.7% |
| **Man vs. Woman** | | | | | | | |
| 1.65  (1.52; 1.8) | > 99.9% | 2.03  (1.86; 2.21) | > 99.9% | 2.79  (2.49; 3.13) | > 99.9% | 4.6  (3.57; 5.98) | > 99.9% |
| **Myocardial infarction, angina pectoris, atrial fibrillation, heart failure, or heart valve disease** | | | | | | | |
| 1.0  (0.93; 1.06) | 51.9% | 0.97  (0.85; 1.06) | 74.0% | 0.97  (0.81; 1.12) | 64.9% | 0.95  (0.63; 1.23) | 66.3% |
| **CABG, PCI intervention, peripheral artery disease intervention, or aortic intervention** | | | | | | | |
| 1.0  (0.92; 1.1) | 50.7% | 0.99  (0.83; 1.15) | 57.4% | 1.06  (0.88; 1.47) | 72.6% | 0.96  (0.47; 1.37) | 62.0% |
| **Stroke** | | | | | | | |
| 1.0  (0.94; 1.14) | 56.8% | 0.97  (0.79; 1.11) | 67.6% | 1.01  (0.81; 1.28) | 54.3% | 0.97  (0.51; 1.4) | 58.0% |
| **Hypertension** | | | | | | | |
| 1.06  (0.99; 1.24) | 87.0% | 1.3  (1.17; 1.49) | > 99.9% | 1.32  (1.14; 1.56) | > 99.9% | 1.89  (1.39; 2.53) | > 99.9% |
| **Hyperlipidemia** | | | | | | | |
| 0.98  (0.83; 1.02) | 78.8% | 1.02  (0.92; 1.13) | 69.4% | 1.35  (1.13; 1.58) | 99.9% | 1.12  (0.9; 1.57) | 83.0% |
| **COPD, chronic bronchitis, emphysema, tuberculosis, or other lung diseases** | | | | | | | |
| 1.0  (0.85; 1.06) | 56.1% | 1.34  (1.04; 1.67) | 99.1% | 2.51  (1.89; 3.23) | > 99.9% | 3.36  (1.86; 5.52) | > 99.9% |
| **Asthma** | | | | | | | |
| 1.0  (0.94; 1.05) | 52.5% | 1.04  (0.96; 1.16) | 84.1% | 0.98  (0.84; 1.11) | 66.6% | 1.0  (0.75; 1.32) | 50.4% |
| **Sleep apnea** | | | | | | | |
| 1.0  (0.94; 1.12) | 53.9% | 1.16  (1.0; 1.39) | 98.0% | 1.64  (1.33; 2.03) | > 99.9% | 1.05  (0.8; 1.57) | 66.1% |
| **Celiac disease** | | | | | | | |
| 1.0  (0.86; 1.08) | 55.2% | 0.98  (0.76; 1.16) | 60.4% | 0.94  (0.5; 1.19) | 70.7% | 0.98  (0.37; 1.61) | 56.0% |
| **Crohn’s disease or ulcerative colitis** | | | | | | | |
| 1.0  (0.9; 1.08) | 52.2% | 1.06  (0.93; 1.37) | 77.7% | 1.02  (0.82; 1.38) | 60.6% | 1.0  (0.56; 1.62) | 50.8% |
| **Rheumatic disease** | | | | | | | |
| 1.0  (0.96; 1.12) | 59.2% | 1.03  (0.94; 1.2) | 74.0% | 1.0  (0.83; 1.18) | 52.4% | 0.98  (0.62; 1.34) | 57.7% |
| **Diabetes** | | | | | | | |
| 1.0  (0.89; 1.06) | 55.4% | 1.19  (1.01; 1.41) | 98.6% | 1.05  (0.91; 1.3) | 74.3% | 0.97  (0.61; 1.3) | 60.4% |
| **Cancer** | | | | | | | |
| 1.0  (0.96; 1.11) | 60.7% | 1.0  (0.91; 1.1) | 51.1% | 0.86  (0.68; 1.02) | 95.3% | 1.0  (0.72; 1.37) | 50.3% |
| **Family history of diabetes, any first degree relative** | | | | | | | |
| 1.0  (0.94; 1.04) | 58.0% | 1.17  (1.08; 1.25) | > 99.9% | 1.05  (0.97; 1.18) | 87.1% | 0.81  (0.59; 1.03) | 95.2% |
| **Family history of asthma, any first degree relative** | | | | | | | |
| 1.0  (0.94; 1.04) | 57.5% | 1.1  (1.02; 1.18) | 99.4% | 0.97  (0.87; 1.06) | 75.2% | 1.0  (0.81; 1.24) | 51.2% |
| **Family history of bronchitis, COPD or emphysema, any first degree relative** | | | | | | | |
| 1.0  (0.95; 1.06) | 50.6% | 1.05  (0.98; 1.14) | 89.7% | 1.23  (1.07; 1.41) | > 99.9% | 1.21  (0.96; 1.68) | 92.7% |
| **Family history of myocardial infarction, subject’s parent or sibling** | | | | | | | |
| 1.0  (0.96; 1.05) | 52.9% | 1.04  (0.98; 1.11) | 90.3% | 0.98  (0.89; 1.07) | 66.5% | 0.9  (0.69; 1.08) | 85.0% |
| **Family history of stroke, subject’s parent or sibling** | | | | | | | |
| 1.0  (0.96; 1.04) | 50.3% | 0.96  (0.9; 1.02) | 88.5% | 0.99  (0.9; 1.08) | 60.5% | 1.14  (0.95; 1.49) | 89.6% |
| **Family history of lung cancer, subject’s parent or sibling** | | | | | | | |
| 1.0  (0.89; 1.03) | 65.8% | 1.06  (0.97; 1.2) | 88.0% | 1.05  (0.93; 1.24) | 77.0% | 1.13  (0.88; 1.69) | 81.6% |
| ^a^ Median of the posterior distribution with 95% compatibility intervals (CI) defined by 2.5% and 97.5% percentiles of the posterior distribution.  ^b^ The proportion of the posterior distribution that is above or below the null in the direction of the median. | | | | | | | |
